# Leptin Acts Independently of Food Intake to Modulate Gut Microbial Composition in Male Mice

**DOI:** 10.1210/en.2013-1085

**Published:** 2014-01-03

**Authors:** Michael W. Rajala, Christa M. Patterson, Judith S. Opp, Susan K. Foltin, Vincent B. Young, Martin G. Myers

**Affiliations:** Department of Internal Medicine, Division of Gastroenterology (M.W.R.), Division of Metabolism, Endocrinology and Diabetes (C.M.P., M.G.M.) and Division of Infectious Disease (J.S.O., S.K.F., V.B.Y.). Department of Microbiology and Immunology (V.B.Y.), Department of Molecular and Integrative Physiology (M.G.M.), and Neuroscience Program (M.G.M.), University of Michigan, Ann Arbor, Michigan

## Abstract

Shifts in the composition of gut bacterial populations can alter host metabolism and may contribute to the pathogenesis of metabolic disorders, including obesity. Mice deficient in leptin action are obese with altered microbiota and increased susceptibility to certain intestinal pathogens. Because antimicrobial peptides (AMPs) secreted by Paneth cells represent a major mechanism by which the host influences the gut microbiome, we examined the mRNA expression of gut AMPs, several of which were decreased in leptin receptor (LepR)-deficient *db/db* mice, suggesting a potential role for AMP modulation of microbiota composition. To address the extent to which the alterations in gut microbiota and AMP mRNA expression in *db/db* mice result from increased food intake vs other defects in leptin action, we examined the effects of pair feeding and gut epithelial LepRb ablation on AMP mRNA expression and microbiota composition. We found that the phylum-level changes in fecal microbial content and AMP gene expression persist in pair-fed *db/db* mice, suggesting that these differences do not stem from hyperphagia alone. In addition, despite recent evidence to support a role for intestinal epithelial LepRb signaling in pathogen susceptibility, ablation of LepRb from the intestinal epithelium fails to alter body weight, composition of the microbiota, or AMP expression, suggesting a role for LepRb elsewhere for this regulation. Indeed, gut LepRb cells are not epithelial but rather constitute a previously uncharacterized population of perivascular cells within the intestinal submucosa. Overall, our data reveal a role for LepRb signaling extrinsic to the intestinal epithelium and independent of food intake in the control of the gut microbiome.

The mechanisms by which the intestinal epithelium interacts with the indigenous gut microbiota to maintain a healthy equilibrium capable of tolerating commensal bacteria while swiftly responding to pathogens are not well understood (1). Dynamic interactions between gut microbes and the host modulate gut cellular proliferation, including the production of secretory cells and gut-associated immune cells (1). Dysregulation of the host-microbiome interaction may contribute to the pathogenesis of systemic metabolic disorders such as obesity (2, 3), metabolic syndrome (4), and cardiovascular disease (5). Shifts in fecal microbial populations correlate with obesity in both mice and humans, suggesting that certain bacterial constituents may modulate the susceptibility or response to weight gain (2, 6). One of the mechanisms by which gut bacteria may influence the host is through fermentation of otherwise indigestible dietary nutrients, rendering them available for host absorption, or through the generation of metabolites that modulate host biology (2, 7). Indeed, the absence of microbes in germ-free animals decreases caloric uptake from the diet and prevents diet-induced obesity. Reintroduction of gut microbes into germ-free mice increases susceptibility to diet-induced obesity (8) and increases adiposity (7, 9).

Leptin is an adipose-derived hormone that circulates in proportion to fat mass/energy stores and controls appetite and metabolic rate, along with other processes (eg, immune and neuroendocrine function) that must be regulated in line with energy availability (10, 11). The absence of leptin signaling in leptin-deficient *ob/ob* or leptin receptor (LepRb)-null *db/db* mice promotes hyperphagia and decreased energy expenditure (with consequent obesity). These animals also display alterations in the gut (fecal) microbiome (increased *Firmicutes* relative to *Bacteroidetes*), similar to those of diet-induced obese animals (9, 12, 13). It remains unclear whether compositional changes in the gut microbiome of obese animals are due to altered leptin action resulting from hyperphagia, from physiologic changes associated with obesity, or from other leptin actions independent of food intake and adiposity. Interestingly, Duggal et al (14) identified a human polymorphism that alters a single amino acid in the extracellular domain of LepRb, which is associated with increased susceptibility to *Entamoeba histolytica*; knock-in of the risk allele in mice produced metabolically normal lean animals with increased susceptibility to *E. histolytica* (15). Thus, these studies suggested a role for leptin signaling in the intestinal epithelium in the defense against gut pathogens, suggesting the possibility that gut epithelial leptin signaling might also shape the community structure of the gut microbiome.

In addition to extrinsic factors (such as diet) that shape gut microbial composition (9), properties intrinsic to the host alter the composition of the commensal bacteria (16). The role of the host in regulating microbiota composition in the context of obesity was established by researchers in the Gewirtz laboratory, who showed that deficiency of host Toll-like receptor 5 signaling results in altered microbiota and a metabolic syndrome phenotype (4). Within the small intestine, Paneth cells produce and secrete into the gut antimicrobial peptides (AMPs) (17), such as α-defensins, with expression restricted to Paneth cells only in mice, as well as others. AMPs not only defend against enteric pathogens but also have the capacity to alter the composition of commensal microbes (18). Mice lacking the enzyme (matrix metalloproteinase 7) that is required for the processing and activation of defensin propeptides and therefore are deficient in defensin activation demonstrate an altered gut microbial composition with increased fecal *Firmicutes* and decreased *Bacteroidetes* relative to those in controls. Reciprocally, transgenic mice overexpressing a human α-defensin in Paneth cells demonstrate decreased fecal *Firmicutes* and increased *Bacteroidetes* compared with those in controls (18). Thus, the production of active α-defensins by Paneth cells can alter the composition of the gut flora; the factors that modulate α-defensin production under normal physiologic conditions remain poorly defined, however.

Given the important role for leptin in controlling diverse processes ranging from metabolism to immunity, we hypothesized that leptin action might modulate bacterial populations within the gut independently of feeding, potentially by controlling the expression of gut AMPs. We furthermore speculated that intestinal epithelial LepRb expression could contribute to this regulation, consistent with its role in the defense against *E. histolytica*.

## Materials and Methods

### Experimental animals

Male and female *db*/+ mice were purchased from The Jackson Laboratory for breeding and housed under specific pathogen–free conditions. Male wild-type and *db/db* offspring were group housed for 4 weeks and then weaned into individual cages for 4 weeks to prevent coprophagy. Intestinal epithelial leptin receptor knockouts were generated by crossing *Lepr^fl/fl^* mice on the mixed C57;FVB background (gift of Streamson Chua) (19) with transgenic mice with a villin-specific (ie, intestinal epithelial cell [IEC]–specific) expression of cre (*V*^*cre*+/−^; gift of Deborah Gumucio) (20) to generate mice with deletion of exon 17 of LepRb (critical for intracellular signaling), in IEC lineage (*V*^*cre*+/−^*-Lepr^FF^*, termed *IEC-Lepr^KO^*) as well as sib-pair controls including *V*^*cre*−/−^*Lepr^FF^*, *V*^*cre*+/−^*Lepr^wt^*, and *V*^*cre*−/−^*Lepr^wt^* animals. Tissue-specific knockout was confirmed by standard PCR using primers that flank both LepRΔ/Δ (226 bp) and LepRF/F (643 bp): forward 5-AATGAAAAAGTTGTTTTGGGACGA and reverse 5′-CAGGCTTCAGAACATGAACACAACAAC (19). Mice were reared for 4 weeks and then individually housed for 4 weeks before sacrifice. LepRb^tomato^ mice were generated by crossing LepRb-cre mice (as described previously) (21) with B6.Cg-*Gt(22)26Sort^m9(CAG-tdTomato)Hze^*/J mice purchased from The Jackson Laboratory. Food and sterile water were given ad libitum except for pair-feeding experiments in which daily intake of chow by wild-type animals was measured, and an equal quantity of food was given to the pair-fed animals. All care and procedures for mice were in accordance with the guidelines and approval of the University of Michigan Committee on Use and Care of Animals.

### Gene expression analysis

For AMP analysis, mice were asphyxiated with CO_2_, and 1 cm of distal ileum was removed for RNA isolation, snap-frozen, and stored at −80°C. For epithelial analysis, 3 cm of jejunum was dissected, opened longitudinally, rinsed with PBS (Ca^2+^Mg^2+^-free), and incubated with 30 mM EDTA and 0.5 mM dithiothreitol for 30 minutes at 37°C with intermittent agitation and the sloughed epithelium (epithelium) and the remaining tissue (deepithelized) were separated. RNA for all samples was prepared using an RNeasy isolation kit (QIAGEN) with treatment of on-column RNase-free DNase treatment (QIAGEN). cDNA was made using the SuperScript II First Strand Synthesis System for RT-PCR (Invitrogen). Expression analysis data was generated using quantitative PCR (Applied Biosystems 7500). Relative expression to GAPDH was calculated using 2^−ΔΔCt^. The following TaqMan assays were purchased from Applied Biosystems: Mm02524428_g1 (defa1), Mm00655850_m1 (defcr-r), Mm00657323_m1 (lyz1), Mm00440616_g1 (reg3β), Mm00441127_m1 (reg3γ), Mm01265583_m1 (LepRb), and 4308313 (TaqMan Rodent GAPDH). In addition, primer/probes were synthesized by Integrated DNA Technologies DNA a-def 5: FAM/TGCTGCAGCTGAATATGCAGATGACAAA/3IABkFQ/GGACCTGCAGAAATCTTTTTTTAACTT (forward) and CTCAGAGCCGATGGTTGTCA (reverse).

### Microbiome analysis

The distal-most fecal pellet was isolated from a mouse immediately after euthanasia by CO2 asphyxiation. Samples were placed in an UltraClean Fecal DNA tube (12811–100DBTD; MO BIO Laboratories), snap-frozen in liquid nitrogen, and stored at −80°C. Total genomic DNA was extracted as follows: 500 μL of MagNa Pure Bacteria Lysis Buffer (046569180001; Roche) was added to the sample, which was promptly placed in a Mini-BeadBeater (BioSpec Products) for 2 minutes on full speed. The sample was centrifuged briefly before addition of 40 μL of 20 mg/mL proteinase K (19133; QIAGEN), and incubated at 65°C for 10 minutes. After digestion with proteinase K, the sample was bead beaten for 1 minute and heat inactivated by incubation at 95°C for 10 minutes. Total DNA was extracted using a MagNa Pure Nucleic Isolation kit (03730964001; Roche) as per the manufacturer's protocol.

The 16S rRNA was sequenced as described previously (23). In brief, the 16S rRNA encoding gene was amplified by PCR using the National Institutes of Health HMP protocol (http://www.hmpdacc.org/doc/HMP_MDG_454_16S_Protocol.pdf) for the V3–V5 hypervariable regions with primer 357F (5′-CCTACGGGAGGCAGCAG-3′) and with a B adaptor (5′-CCTATCCCCTGTGTGCCTTGGCAGTCTCAG-3′) and primer 926R (5′-CCGTCAATTCMTTTRAGT-3′) with a unique barcode to identify the specimen and an A adaptor (5′-CCATCTCATCCCTGCGTGTCTCCGACTCAG-3′). Primary PCRs were performed using 1 μL of DNA and 0.2 μM concentrations of each primer (A and B) in the presence of AccuPrime *Taq* (DNA Polymerase, High Fidelity) and Buffer II (12346–094; Invitrogen) under the thermal cycler conditions of an initial melting temperature of 95°C for 2 minutes, followed by 30 cycles of 95°C for 20 seconds, 50°C for 30 seconds, and 72°C for 5 minutes, ending with a hold temperature of 4°C. A small sample of each primary PCR product was verified to consist of the expected 660-bp band fragment on an E-Gel (2% agarose) containing SYBR Safe (G7208–02; Invitrogen). Subsequently each sample was purified using AMPure beads (A63881; Beckman Coulter Genomics) followed by quantification by a PicoGreen dsDNA quantitation assay (P11496; Invitrogen) in duplicate. Samples were pooled, normalizing for the number of molecules before undergoing a final round of purification using AMPure beads. Quality control of the pooled library was monitored by first observing a single band on an Agilent bioanalyzer using a High Sensitivity DNA Kit (5067–4627; Agilent) for double-stranded DNA and last by determining molecules per microliter using quantitative PCR techniques (KK4821; KAPA Biosystems). Pyrosequencing was performed on the finished library using a GS Junior Titanium platform (Lib-L; Roche), allowing for 0.4 copy per bead and a load capacity of 500 000 beads per picotiter plate (see Roche protocols for emPCR and sequencing method manuals). Images were processed using Roche's algorithms for amplicons.

### Submucosal cell isolation

Mice were asphyxiated with CO_2_, and 3 cm of small or large intestine were removed, opened longitudinally, and rinsed by shaking with 10 mL of Hanks' balanced salt solution (HBSS)-10 mM EDTA-2% fetal calf serum (FCS)-1× antibiotic/antimycotic (GIBCO) with frequent buffer changes until tissue cleared of stool residue. Tissue was then incubated at 37°C for 30 minutes with frequent shaking (∼every 5 minutes) to remove epithelium. In some experiments, the sloughed epithelium was spun down, and the RNA was isolated from the epithelium and deepithelialized intestinal tissue for comparative analysis. In cell sorting experiments, the epithelium was discarded and the deepithelialized tissue was then rinsed and incubated with RPMI 1640 medium-5% FCS-HEPES-antibiotic/antimycotic at 37°C for 10 minutes, repeated 1 time. Tissue was then minced into ∼1-mm cubes, resuspended in RPMI 1640 medium-5% FCS-HEPES-antibiotic/antimycotic-Liberase DH (0.5 μg/mL; Roche) and incubated at 37°C with pipetting for 20 seconds every 5 minutes for a total of 30 minutes or until tissue dissociated as judged by examination under a fluorescent microscope. The cells were spun down at 500 × *g* for 5 minutes, rinsed 3 times with HBSS-1% EDTA, resuspended with 4 mL of HBSS-1% EDTA, and filtered through a 70-μm cell strainer (BD Falcon). 4,6-Diamidino-2-phenylindole (DAPI) was added (0.2 μg/mL) to the single-cell suspension, and cells were incubated on ice until sorting (about 30 minutes). Both DAPI-negative/Tomato-positive cells (“positive sort”) as well as DAPI-negative/Tomato-negative cells (“negative sort”) were sorted directly into RLT buffer (QIAGEN) by fluorescence activated cell sorting (FACS) in the core facility at the University of Michigan. A quantity of 100 μL of unsorted cells was saved and added directly to RLT buffer for the “unsorted” sample. RNA was isolated from all samples according to QIAGEN RNeasy protocol.

### Immunohistochemistry

Mice were euthanized, and the small bowel and colon were removed, rinsed by flushing with ice-cold PBS until clear, and then cut longitudinally on Whatman paper with the epithelial side up. The tissue and paper were submerged in 10% PBS-buffered formalin and fixed 2 hours before switching to 30% sucrose in PBS at 4°C. On the following day, the tissue was either “jelly-rolled” for examination of the whole intestine, or 2-cm segments from the different intestinal segments (duodenum–proximal-most small bowel, ileum–distal-most small bowel, and the jejunum equidistant between the two, colon and cecum) were embedded and then snap-frozen in O.C.T. (Tissue-Tek). Tissue blocks were cryosectioned (6 μm) and mounted on slides. Slides were allowed to come to room temperature for 5 minutes and then were rinsed 3 times with Tris-buffered saline/Tween 20 (TBST) and blocked with 5% BSA-TBST for 1 hour. Sections were then incubated with rabbit anti-LyVe-1 diluted 1 μg/mL, ab14917; Abcam) and rat anti-CD31 (diluted 1:50; BD Pharmingen) in TBST overnight at 4°C in a humid chamber, rinsed 5 times for 5 minutes in TBST and incubated with Alexa Fluor 350 anti-rabbit (1:200; Molecular Probes) and Alexa Fluor 488 anti-rat (1:200; Molecular Probes) in block for 1 hour before rinsing 5 times with TBST for 5 minutes. Antibodies were not required to detect tdTomato expressed in reporter mice.

### Statistical analysis

The Student *t* test was used to compare 2 test groups. Two-way ANOVA with a Bonferroni correction post-testing was used for comparisons of multiple groups. Differences were considered significant for a value of *P* < .05.

## Results

### Altered expression of antimicrobial peptides in *db/db* mice

**Figure 1.**
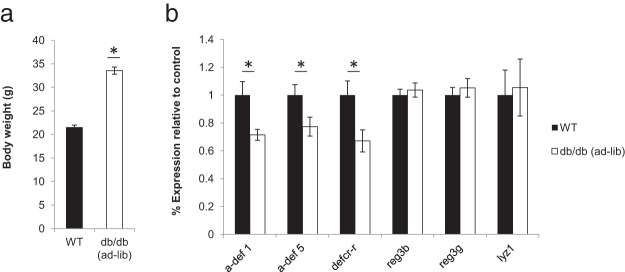
Quantitative expression analysis of Paneth cell–specific antimicrobial peptides in ad libitum–fed *db/db* mice compared with that in wild-type (WT) animals. A, Average body weight (grams) of wild-type and *db/db* mice at 8 weeks of age. B, Percent mRNA expression of antimicrobial peptides in the distal ileum relative to the control (GAPDH) for both wild-type and *db/db* animals. Graphed data represent average values ± SEM. n = 13 for wild-type and n = 10 for *db/db* mice. Statistical analysis with the Student *t* test: *, *P* < .05

Both *ob/ob* and *db/db* mice have altered fecal microbial composition (increased *Firmicutes* relative to *Bacteroidetes*), similar to those of other obese animals (6, 9, 13). Because decreased AMP activity can shift gut microbial composition (also increasing fecal *Firmicutes* relative to *Bacteroidetes*) (18), we examined gut AMP expression in ad libitum–fed (obese) *db/db* animals (Figure 1A) to determine whether changes in AMPs could contribute to the gut microbial alterations in these mice. We performed quantitative PCR on RNA extracted from the distal ileum of *db/db* and littermate control animals to determine relative mRNA expression for several classes of AMPs including α-defensins (α-defensin 1, α-defensin 5, and defcr-r) and lysozyme (lyz1) whose expression is restricted to Paneth cells as well as C-lectins (reg3β and reg3γ) that have expression predominantly in Paneth cells but also in enterocytes (Figure 1B). This analysis revealed an ∼20% to 30% reduction in the mRNA expression of Paneth cell–specific AMPs including α-defensin 1, α-defensin 5, and defcr-r in the *db/db* animals (Figure 1B). Other AMPs tested were not significantly different between the 2 groups.

### Altered microbiota and AMP expression in *db/db* mice are independent of increased food intake

**Figure 2.**
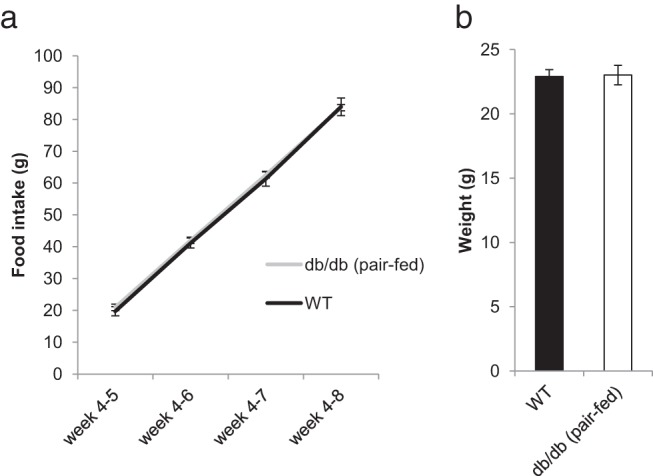
Cumulative food intake (grams) from weeks 4 to 8 (A) and average body weight (grams) at 8 weeks of age (B) did not differ between wild-type (WT) and pair-fed *db/db* mice. n = 13 for wild-type and n = 20 for pair-fed *db/db* mice.

**Figure 3.**
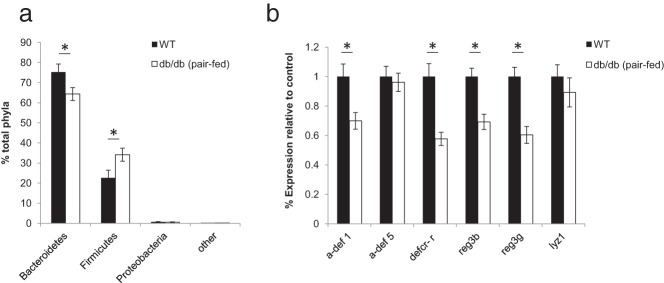
Altered microbiota composition and expression of antimicrobial peptides in pair-fed *db/db* animals compared with those in wild-type (WT) mice. A, Comparison of fecal microbiota populations by 16S rRNA-encoding gene sequencing. B, Percent relative mRNA expression of antimicrobial peptides in the distal ileum (normalized to GAPDH). Graphed data represent average values ± SEM. n = 13 for wild-type and n = 20 for pair fed *db/db* mice. Statistical analysis with two-way ANOVA (A) and the Student *t* test (B): *, *P* < .05

To address whether the changes in microbial composition or AMP expression observed in *db/db* mice were simply due to increased food intake and/or represented the sequelae of obesity, we controlled for these conditions by pair feeding (5) *db/db* mice to the daily food intake of their ad libitum–fed wild-type sib controls (Figure 2A). All mice were bred from common *db*/+ founders and then singly housed after 4 weeks of age to reduce alterations in microbiota composition caused by maternal, environmental, or strain variation. As expected after pair-feeding, the average weights of the wild-type and pair-fed *db/db* mice were not significantly different at 8 weeks of age (Figure 2B). Analysis of the fecal microbiota by 16S rRNA-encoding gene sequencing in these animals revealed altered microbial composition in the pair-fed *db/db* animals, with an increased percentage of *Firmicutes* and decreased *Bacteroidetes* relative to those of wild-type animals (Figure 3A). These results are consistent with the notion that hyperphagia is not the primary cause of altered gut microbial composition in *db/db* animals.

To determine whether the observed differences in AMP mRNA expression between ad libitum–fed *db/db* animals persisted in lean pair-fed *db/db* mice, we also analyzed gut AMP expression in these animals. As for the ad libitum–fed mice, the expression of α-defensin 1 and defcr-r were significantly reduced in the lean pair-fed *db/db* animals, whereas lyz1 expression was similar to that of controls (Figure 3B), suggesting that leptin modulates the expression of these AMPs independently of food intake and body weight. In addition, in contrast to ad libitum–fed *db/db* animals in which there was reduced expression of α-defensin 5, expression in the pair-fed *db/db* animals did not differ significantly from that of controls. Similarly, the expression of reg3β and reg3γ was similar between ad libitum–fed *db/db* mice and wild-type mice but was significantly reduced in pair-fed *db/db* mice. These differences suggest that the expression of reg3β and reg3γ is probably regulated by other mechanisms, possibly in response to shifts in microbial populations.

Overall, our data reveal that leptin acts independently of food intake to control the expression of several AMP-encoding genes in the gut epithelium, in addition to gut microbiome composition. These food intake–independent changes presumably stem from other alterations resulting from disrupted leptin action, although the site(s) and mechanism(s) by which leptin modulates gut AMP gene expression and the microbiome remain unclear.

### Leptin modulates metabolism, the gut microbiome, and AMP gene expression independently of intestinal epithelial LepRb

**Figure 4.**
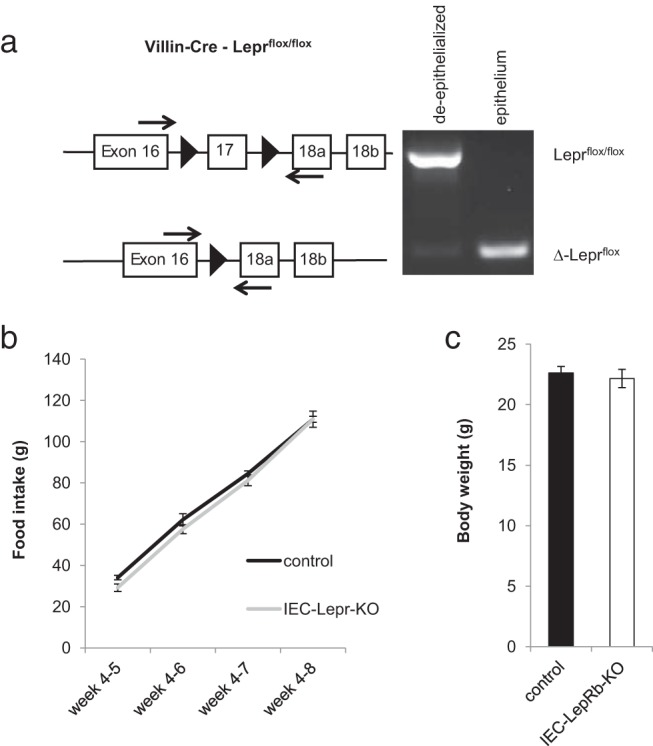
IEC-LeprKO mice. A, Genomic deletion of LepRb by Villin-Cre is specific for intestinal epithelium. B and C, Cumulative food intake (grams) from weeks 4 to 8 (B) and average body weight (grams) at 8 weeks of age (C) did not differ between IEC-LepRb-KO mice and the control cohort. n = 11 for IEC-lepRb-KO and n = 26 for controls. Not significant.

The recent finding that the absence of gut epithelial LepRb in mice confers susceptibility to *E. histolytica* pathogenesis suggested the possibility that gut epithelial leptin action might modulate AMP expression and gut microbial composition. To test this possibility, we generated mice lacking gut epithelial LepRb (*Vil^cre^;Lepr^flox/flox^*, heretofore referred to as IEC-LepRb-KO mice), as described previously, along with littermate controls of several genotype variations to control for any strain variation. As above, these animals were generated by intercrossing offspring from common founders to mitigate against potential genetic and maternal influences on microbiome composition; offspring were group-housed briefly upon weaning before individual housing, also as above. PCR genotyping of DNA extracted from gut epithelium or deepithelialized intestinal tissue confirmed the excision of exon 17 from the epithelium but not the remaining deepithelialized gut tissue (Figure 4A). The faint lepRbΔ signal within the deepithelialized sample probably results from a small amount of residual epithelium not fully sloughed off.

**Figure 5.**
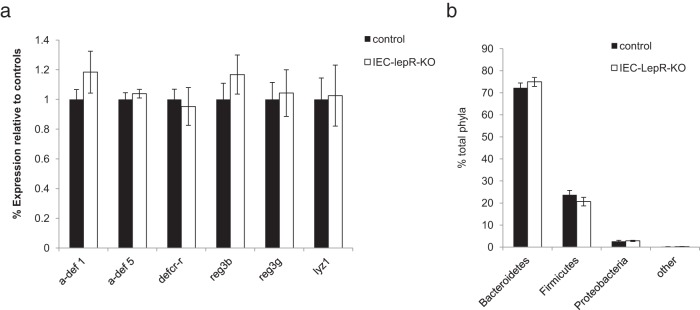
Microbiota composition and expression of antimicrobial peptides do not differ between IEC-Lepr-KO mice and control. A, Percent relative mRNA expression of antimicrobial peptides in the distal ileum (normalized to GAPDH). B, Comparison of fecal microbiota populations (% total phyla) by 16S rRNA-encoding gene sequencing. Graphed data represent average values ± SEM. n = 10 for IEC-LepRb-KO mice and n = 25 for the control group. Statistical analysis with the Student *t* test (A) and two-way ANOVA (B): not significant.

Consistent with the primarily central nervous system–dependent control of food intake and adiposity by leptin, IEC-LepRb-KO mice exhibited food intake and body weight indistinguishable from those of control animals (Figure 4, B and C), suggesting no role for intestinal epithelial LepRb in the control of these parameters. Furthermore, our analysis revealed no difference in fecal microbial composition or AMP gene expression in IEC-LepRb-KO mice compared with that in controls (Figure 5, A and B). These results indicate that intestinal epithelial LepRb signaling does not significantly affect the luminal bacterial composition or gene expression of AMPs in the intestinal epithelium, suggesting that leptin action on other sites must mediate these effects.

### Identification of an uncharacterized submucosal perivascular LepRb^+^ cell population

**Figure 6.**
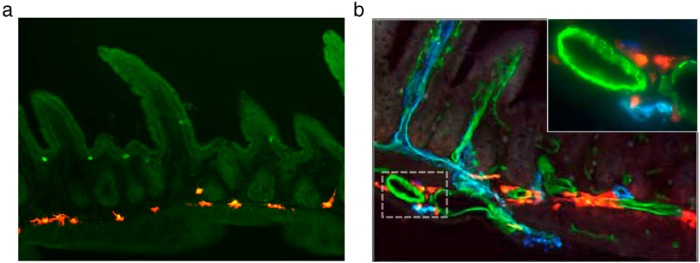
Immunohistochemical analysis of ileum (A) from LepRb-Cre × Rosa26-Tomato reporter mice reveals labeling of a submucosal cell population and absence of epithelial labeling. Costained ileal sections (B) from these mice with antibodies against endothelial (CD31, green) and lymphatic (LyVe-1, blue) markers confirmed perivascular localization (B, inset).

Because of the lack of altered microbiota or AMPs in the IEC-KO mice, we more closely examined whether (and which) gut cells might express LepRb (Figure 5). To visualize gut LepRb cells, we crossed our LepRb-Cre mouse line (which expresses cre recombinase only in cells expressing the long form of the leptin receptor) onto the cre-inducible tdTomato fluorescent protein reporter line to generate LepRb^tomato^ mice with fluorescently labeled LepRb^+^ cells. Immunohistochemical analysis of the small and large bowels of these mice revealed the presence of LepRb^+^/Tomato^+^ cells within the intestinal submucosa (Figure 6A), but no reporter expression within the gut epithelium.

To further characterize these submucosal LepRb^+^ cells, immunohistochemical analysis was used to confirm the presence or absence of colocalization with markers of suspected cell types. There was no colocalization with neuronal (Tuj1, NeuN, ChAT, and nNOS), myeloid (CD45 and CD11b/c), myofibroblast (desmin and smooth muscle actin [SMA]), or pericyte markers (SMA) by immunohistochemical analysis or by flow cytometry (CD11c and MHCII) (data not shown). Endothelial (CD31) and lymphatic (LyVe-1) markers revealed the absence of colocalization with LepRb^+^ cells, but close approximation with the vasculature within the intestinal submucosa (Figure 6B).

### Submucosal perivascular LepRb^+^ cell population is the predominant source of LepRb expression in the intestine

**Figure 7.**
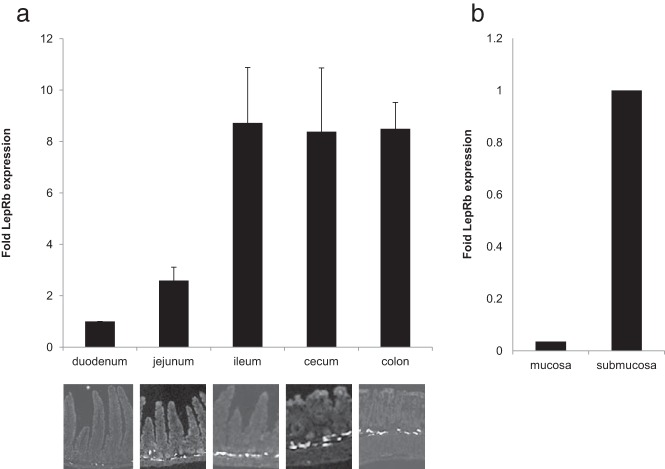
Quantitative expression analysis of LepRb from 1-cm intestinal segments from LepRb-tomato reporter mice reveals a marked increase in LepRb expression relative to that of GAPDH from proximal to distal bowel (A, graph). Concomitantly, the presence of LepRb^+^-tomato fluorescent-labeled cells increases in frequency within the submucosa (A, immunohistochemical analysis). Quantitative analysis of LepRb relative to GAPDH in epithelium vs the remaining deepithelialized ileum (B) revealed lower expression within the epithelium. Graphed data represent average values (A and B) ± SEM (A). n = 3 for each.

Analysis of the entire intestine showed that tomato-positive cells within the submucosa increased in frequency from the proximal to the distal intestine (Figure 7A). To determine whether LepRb mRNA increased in parallel with the immunohistochemical findings, we performed quantitative PCR on RNA extracted from intestinal segments from the duodenum to the colon. In agreement with immunohistochemical findings, LepRb expression also increased >8-fold from duodenum to ileum, after which it remained at a stable plateau throughout the cecum and colon (Figure 7A).

To further investigate the unexpected lack of epithelial labeling, we separated gut epithelium from the remainder of the intestinal tissue (deepithelialized) and compared expression between the two. Consistent with the absence of reporter expression within the epithelium of LepRb^tomato^ mice, LepRb mRNA was >50-fold less abundant in the epithelium (n = 3, *P* = .01) (Figure 7B).

**Figure 8.**
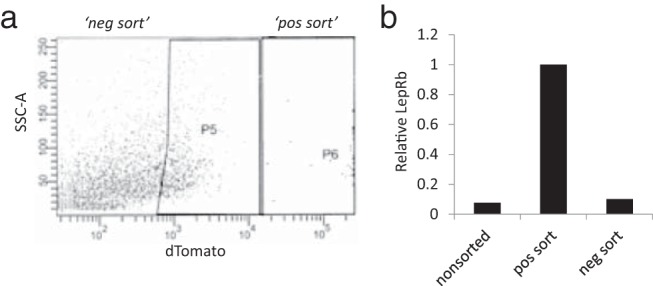
A, FACS of dissociated ileum from LepRb-tomato reporter mice revealed these cells to be 0.25% to 0.5% of the total cell population within the ileum. B, Quantitative analysis of LepRb expression relative to GAPDH in unsorted but dissociated cells, tomato fluorescent “positive” cells and tomato fluorescent “negative” cells confirm that the tomato-positive cells are the predominant source of LepRb mRNA within the intestine. pos, positive; neg, negative.

To confirm that the reporter-positive cells actually express LepRb transcript and account for the predominant source for LepRb expression within the deepithlialized tissue, deepithelialized small intestine was dissociated into single cells by enzymatic digestion and pure populations of reporter-positive cells were isolated by FACS (Figure 8A). The isolated cells accounted for 0.25% to 0.5% of the total cellular population. Quantitative gene expression confirmed that LepRb was expressed within these cells and that expression levels were >10-fold higher (*P* = .0007) in LepRb reporter–positive cells than in either negatively sorted or unsorted dissociated cells, thus confirming that the tomato-positive cells are the predominant LepRb-positive cells in the small and large intestines (Figure 8B).

## Discussion

In this study, we provide data that support a role for LepRb signaling extrinsic to the intestinal epithelium and independent of food intake in the host regulation of microbiota composition. We also identify a previously unknown population of perivascular LepRb^+^ cells within the intestinal submucosa.

The pair-fed *db/db* mice had weights, housing conditions, diet composition, and founders that were identical to those of their wild-type sib-pair controls, which allows for comparison of microbiota between cohorts. The gut microbiome of pair-fed LepRb-deficient mice demonstrated a significantly increased ratio of phylum Firmicutes to phylum Bacteroidetes relative to that of wild-type sib pairs, similar to the change associated with ad libitum–fed obese leptin-deficient mice. Thus, leptin signaling influences gut microbial composition independently of food intake and body weight, although the precise mechanism is uncertain.

It has been speculated that an “obese” microbiome might contribute to obesity and metabolic dysfunction (24), and understanding the regulatory factors capable of altering gut microbial composition to a more metabolically favorable state is of great clinical interest. Despite multiple studies, the mechanisms that directly regulate AMPs and other host defense mechanisms remain largely unknown. Because the production of AMPs by the gut epithelium alters gut microbial composition (18), we speculated that changes in the gut microbiome observed in mice deficient in leptin action might stem from altered AMP expression. Consistently, the mRNA expression of several AMPs was decreased in pair-fed mice compared with that in controls. In comparison, there was no change in microbiota composition in the LepRb-IEC-KO mice, nor was there differential expression of AMPs between the cohorts, which is consistent with mutually responsive relationship. In this study, we focused on 3 classes of antimicrobial peptides; the α-defensins, the c-lectins, and lysozyme. Each class of AMPs possesses different mechanisms of action and microbial targets. For example, α-defensins target Gram-positive and Gram-negative bacteria as well as some fungi, viruses, and protozoa and have been shown to have the capacity to alter luminal microbial populations (16). Alternatively, c-lectins (such as reg3γ) are bacteriocidal for certain Gram-positive bacteria and appear to be especially important to control invasion within the adjacent epithelial mucous layer (16). Although we did note decreased c-lectin (reg3β and reg3γ) expression in pair-fed *db/db* mice compared with that of controls, these differences are not likely to be responsible for the observed changes in luminal bacterial populations, because the absence of reg3γ has been shown not to alter luminal microbiota composition (25). Further, the expression of reg3γ is strongly induced by bacteria, and expression is essentially absent in gnotobiotic animals, suggestive of a more defensive regulatory role. Conversely, overexpression of α-defensins or the loss of α-defensin propeptide activation alters luminal bacterial composition, consistent with the reduced α-defensins and changes in gut microbiota we observed in the pair-fed *db/db* animals. Because the expression of lysozyme and many α-defensins is independent of microbiota, it follows that other factors would play a role in their regulation.

Furthermore, a leptin receptor polymorphism is associated with increased susceptibility to *E. histolytica* infection, both in humans (14) and in mice (15), suggesting a significant role for LepRb in the host response to gut organisms. Eliminating LepRb from the gut epithelium failed to change the gut microbial composition, metabolic parameters, or the expression of AMPs, however, suggesting that the effects of leptin signaling on AMP expression and microbiota composition are mediated independently of any direct effects of leptin on the gut epithelium. The discrepancy between susceptibility to *E. histolytica* infection related to epithelial knockout of the leptin receptor and our inability to detect significant LepRb mRNA expression in the epithelium might be due to the fact that we analyzed the epithelium only for the long isoform of the receptor (LepRb). Indeed, the mutation associated with increased *E. histolytica* susceptibility is in the extracellular domain of the leptin receptor and is present in all isoforms. Interestingly, this mutation also lies outside of the leptin receptor binding region of the extracellular domain, suggesting a potential leptin-independent function for the mutation (and the receptor).

Although diet has also been shown to have a significant influence on gut microbial composition (9), our results from the pair-fed *db/db* mice argue against hyperphagia alone being the cause of the “obese microbiota” observed in these mice. Herein we have shown that the genetic ablation of LepRb from the intestinal epithelium did not affect gut microbiota, AMP expression, or metabolic parameters, probably because of the lack of significant LepRb expression within the epithelium as suggested by LepRb-Cre reporter studies and quantitative mRNA expression analysis of intestinal epithelium.

We have identified a population of perivascular LepRb cells that provide a potential alternate explanation for the observed effects of leptin signaling on AMP expression and gut microbial content. The increasing density of these cells from the proximal to the distal bowel parallels the increasing bacterial load within the gut lumen, a finding that would be in line with leptin signaling playing a role in the interaction between the host and the microbiota. Further, in support of these cells being the primary cell type expressing LepRb mRNA within the gut, analysis of total LepRb mRNA expression in intestinal segments showed a marked increase in the distal ileum and colon compared with that in the duodenum, a finding that mirrors the increase in density of LepRb^+^/tomato^+^ reporter cells.

Given their location near vascular structures within the submucosa, we examined whether these cells could be either myofibroblasts, pericytes, or neurons or of myeloid lineage. We found no overlap between the submucosal LepRb cells and neuronal (Tuj1, NeuN, ChAT, and nNOS), myeloid (CD45 and CD11b/c), endothelial (CD31), lymphatic (LyVe-1), or myofibroblast (desmin and SMA) markers, however. The close association of LepRb cells with the vasculature suggests that they may represent some type of pericyte, although it would be an atypical pericyte that does not express desmin and is SMA-negative and also restricted to larger submucosal vessels. Alternatively, a recent report by Ding et al (26) describes a population of LepRb^+^ perivascular/perisinusodal cells within the bone marrow that provide a key niche component in maintaining hematopoietic stem cells; the role of LepRb signaling in these cells also remains undefined, however. It is therefore possible that the intestinal submucosal LepRb^+^ perivascular cells identified here could support a stem cell line involved in host defense.

Despite our findings, the precise mechanism by which leptin affects AMP expression remains unclear. Similarly, whether leptin signaling influences microbiota composition by altering AMP expression or whether leptin signaling influences microbiota by another mechanism, which in turn induces expression of specific AMPs is also unclear. Given the established role for leptin in both innate and acquired immunity (27), alternative mechanisms by which leptin influences gut microbiota may be through altered cytokine expression or release and/or changes in immune cell populations in *db/db* animals. For example, defective leptin action decreases the production of proinflammatory cytokines (TNFα, IFN-γ, IL-1β, IL-6, IL-18, and others) (27) and reduces (among others) invariant natural killer T cells that recognize lipid antigens presented by CD1d, a major histocompatability class I–related protein that is constitutively expressed in IECs and regulates lysozyme secretion and gut microbial composition (28).

In conclusion, our data reveal a role for LepRb signaling extrinsic to the intestinal epithelium and independent of food intake in the host regulation of gut microbiota composition. Furthermore, there exists a population of LepRb-positive perivascular cells within the intestinal submucosa; these cells increase in frequency proceeding distally in the intestine. The distribution of these cells throughout the gut is consistent with a potential role in host defense, but more work will be required to define their physiologic function. Similarly, additional work will be required to understand the mechanisms by which leptin controls gut microbiota and AMP expression.
